# Retraction Note: Dietary intake and incidence risk of idiopathic pulmonary fibrosis: a Mendelian randomization study

**DOI:** 10.1186/s12890-024-02963-5

**Published:** 2024-03-15

**Authors:** Yilin Zhang, Yihong Gan, Hong Zhang

**Affiliations:** 1 The First School of Clinical Medicine, Zhejiang Chinese Medical University, Hangzhou, China; 2 The First Affiliated Hospital of Zhejiang Chinese Medical University, Hangzhou, China

**Retraction Note: BMC Pulmonary Medicine (2023) 23:376**

10.1186/s12890-023-02673-4

The Editor has retracted this article because, owing to an administration error, it was published before the peer review process had been undertaken. Post-publication peer review has concluded that the article does not present an a priori hypothesis and the methodology is not adequately described. In addition, there is insufficient evidence for a causal relationship between the intake of oily fish and dried fruit and idiopathic pulmonary fibrosis. The authors have been invited to submit a new manuscript for peer review. Hong Zhang has not explicitly stated whether they agree with this retraction. Yilin Zhang and Yihong Gan disagree with this retraction. The Publisher apologies to the authors for the administrative error.

